# Experimental and Clinical Advances in Counteracting Progression of Solid Cancers

**DOI:** 10.3390/cancers15071956

**Published:** 2023-03-24

**Authors:** Andrea Nicolini

**Affiliations:** Department of Oncology, Transplantations and New Technologies in Medicine, University of Pisa, 56126 Pisa, Italy; andrea.nicolini@med.unipi.it

In recent decades, impressing technological developments have significantly advanced our understanding of cancer. In particular, a huge amount of studies and clinical trials have provided new findings for most solid tumors, and their contributions have enriched the operating field of oncologists. This Special Issue offers a useful reference for readers, who will find a detailed update on various subjects spanning different cancers, such as as Malignant Pleural Mesothelioma (MPM), breast cancer, penile cancer, salivary gland neoplasms (SGN), pediatric solid tumors, thyroid cancer, as well as solid tumors in general. The first paper is a relevant study by Dell’Anno, I. et al., that reports on an intriguing proposal for repurposing drugs to treat Malignant Pleural Mesothelioma cancer patients [1]. The experiments carried out in vitro and in vivo comprehensively showed the anti-proliferative efficacy of the investigated drugs. In particular cephalomannine, ouabain, thonzonium bromide, and emetine seem to represent novel drugs that are capable of significantly increasing the therapeutic armamentarium to fight MPM. This is also of great importance considering that, so far, MPM has been characterized by the lack of effective therapy. The two following papers by Frayen, G. et al. and by Wazir, V. et al. focus on diagnostic advances in solid tumors and breast cancer, respectively [2,3]. The former presents data for the validation of a targeted panel for large mutational and biomarkers analysis based on Next Generation Sequencing (NGS). Namely, the assessed hybrid-capture-based comprehensive Trusight Oncology (TSO500) assay showed >99% accuracy in detecting the main DNA or RNA genetic aberrations, starting with only 80 mg DNA or 40 mg RNA obtained by formalin-fixed and paraffin-embedded (FFPE) samples. Additionally, this genomic profiling assay was successfully validated in a clinical diagnostic setting of broad tumor screening by using a cohort of 624 diagnostic samples. The latter study presents and discusses the findings of a study on a novel tool as a wireless alternative for the localization of non-palpable breast lesions before excision. The used method was the SAVI SCOUT system, which was investigated at the London Breast Institute. The study reports some principal parameters with attractive results, such as a mean distance from the lesion of 1.1 mm; a median operating time for malignancy excision and for diagnostic excision of 28 and 17 min, respectively; and an incidence of reflector migration of 0%. More importantly, no concomitant significant surgical complication occurred, and therefore the authors conclude that the proposed method “is an effective and time-efficient alternative to wire-guided localization (WGL) with excellent physicians and patients acceptance”. The role of the PI3K/mTOR/AKT pathway in relatively rare male cancers such as penile cancer is addressed in the review article by Thomas, A. et al. [4]. Tissue microarray analysis and immune-histochemical staining were performed with antibodies against some different molecules of this pathway; their protein expression was correlated with clinic-pathological characteristics as well as clinical outcome. Treatment with the pan-AKT inhibitor capivasertib was also evaluated in treatment-naive PeCa cell lines by an analysis of cell viability and chemotaxis. AKT was the most promising biomarker and increased expression of AKT significantly prevailed in high-grade tumors and was an independent predictor of worse overall survival (OS) and disease-specific survival (DSS) in the multivariate regression analysis. Accordingly, capivasertib in PeCa cell lines induced a significant downregulation of both total AKT and pAKT as well as decreased cell viability and chemotaxis. In the overview by Iyer, J. et al., immune-histochemistry, fluorescent in situ hybridization, and next-generation sequencing are the reported innovative approaches that may assist in identifying various salivary gland neoplasms (SGN); these still represent a diagnostic dilemma due to their different histo-morphological appearances and various histo-pathological subtypes with overlapping features [5]. Although surgical removal remains the principal therapeutic intervention for most SGNs, the overview offers readers a comprehensive look at other common and more recent modalities for their treatment. Moreover, the main criteria to be applied are considered and recent diagnostic protocols that may make the identification and management of SGN easier are highlighted. Another review deals with a significant challenge in oncology: the treatment of relapsed and refractory pediatric solid tumors [6]. The primary focus is on the effective use of natural killer (NK) cells. In particular, although the administration of allogeneic NK cells is a potential therapeutic option with the advantage of not causing graft-versus-host disease, so far, the clinical efficacy of adoptive NK cell therapy has been disappointing. Thus, the review sheds light on and examines a detailed array of the biological pathways within NK cells that can be used to develop “next generation” NK cell therapies. Combining NK cells with other immune therapies, cytokines, checkpoint inhibition, and engineering NK cells with chimeric antigen receptors (CARs) are among the current approaches to optimize the NK cell antitumor response. The successive review article entitled “molecular genetics of follicular derived thyroid cancer” examines the genetic landscape of follicular-derived thyroid tumors and differences across histological subtypes [7]. Molecular testing is the proposed tool to provide insightful information to refine the risk stratification of follicular thyroid tumors. Moreover, in the presence of molecular alterations, the same diagnostic tool will guide the therapeutic strategies for patients with advanced tumors who do not respond to standard treatment. The second to last review focuses on the role played by exosomes in cancer progression, mainly through cross-talk within the tumor microenvironment (TME) and their usefulness for cancer treatment [8]. This issue has raised increasing interest in recent years, becoming a “hot“ topic in the scientific literature and among researchers in oncology. The start of the article, following a detailed description of the biogenesis of exosomes, is entirely devoted to the bioactive molecules that they export as exosomal cargo; these can function as biomarkers in diagnosis or play a relevant role in tumor microenvironment (TME) remodeling and pre-metastatic niche formation, in modulating the immune system, and in promoting apoptosis, cancer development, and growth. In the following part, the potential role of exosomes for cancer therapy, particularly their use as carriers of natural substances and drugs with anticancer properties, is discussed. Finally, the main aspects and the rationale of the intriguing therapeutic proposal of the use of exosomes as biological reprogrammers of cancer cells is highlighted. The last paper by Swarnkar, P.K. et al. offers a systematic review where the PubMed, Cochrane, and Google Scholar databases were the main sources of data on more than 3000 studied patients [9]. The aim was to determine the false-negative rate (FNR) of both marked lymph node biopsy (MLNB) alone, and of targeted axillary dissection (TAD) (MLNB plus SLNB), compared with the gold standard of complete axillary lymph node dissection (cALND).

In a pooled analysis of 366 patients from nine studies that met the inclusion criteria, the FNR of MLNB alone was not significantly different from that observed in 521 patients from 13 studies where SLNB was carried out in addition to MLNB (TAD); in fact, the FNR was 6.28% and 5.18%, respectively (*p* = 0.48). In another analysis, a pooled success rate of 90.0% for the identification and surgical retrieval of the MLN was found. Authors conclude by stating that “the FNR associated with MLNB alone or combined with SLNB is acceptably low and both approaches are highly accurate in staging the axilla in patients with node-positive breast cancer after NACT”; however, soon after, they warn that “further research to confirm the oncological safety of this de-escalation approach of axillary surgery is required”.
